# Understanding Loneliness in Adolescence: A Test of Competing Hypotheses on the Interplay of Extraversion and Neuroticism

**DOI:** 10.3390/ijerph182312412

**Published:** 2021-11-25

**Authors:** Larissa L. Wieczorek, Sarah Humberg, Denis Gerstorf, Jenny Wagner

**Affiliations:** 1 Educational Psychology and Personality Development, University of Hamburg, Von-Melle-Park 5, 20146 Hamburg, Germany; jenny.wagner@uni-hamburg.de; 2 Psychological Diagnostics and Personality Psychology, Institute for Psychology, University of Münster, Fliednerstr. 21, 48149 Münster, Germany; sarah.humberg@wwu.de; 3 Developmental and Educational Psychology, Humboldt University, Rudower Chaussee 18, 12489 Berlin, Germany; denis.gerstorf@hu-berlin.de

**Keywords:** loneliness, personality, adolescence, polynomial regression, information-theoretic approach

## Abstract

Given that adolescents often experience fundamental changes in social relationships, they are considered to be especially prone to loneliness. Meanwhile, theory and research highlight that both extraversion and neuroticism are closely intertwined with individual differences in loneliness. Extant research has explored the linear main effects of these personality traits, yet potential non-linear associations (e.g., exponential effects) and the potential interplay of extraversion and neuroticism (e.g., mutual reinforcement effects) remain unknown. We addressed these open questions using cross-sectional and one-year longitudinal data from two adolescent samples (overall *N* = 583, *M_age_* = 17.57, 60.55% girls) and an information-theoretic approach combined with polynomial regression. Analyses showed little evidence for interaction effects but revealed non-linear effects in addition to the main effects of extraversion and neuroticism on loneliness. For example, the positive cross-sectional association between neuroticism and loneliness was stronger at higher neuroticism levels (i.e., exponential effect). Results differed across loneliness facets in that both traits predicted emotional loneliness, but only extraversion predicted social loneliness. Longitudinal analyses showed that loneliness changes were mainly related to neuroticism. We discuss results in the light of sample differences, elaborate on the importance to differentiate between emotional versus social aspects of loneliness, and outline implications for adolescent development.

## 1. Introduction

Loneliness is defined as the distressing feeling that accompanies the perceived discrepancy between desired and actual quality or quantity of social relationships [1,2]. Although temporary feelings of social isolation reflect a common experience, more chronic feelings of loneliness can have serious consequences for mental and physical health [2,3]. The adolescent years mark a time during which many developmental changes take place, including the transition out of school, identity exploration, and social relationship reorganization [4,5]. Specifically, adolescents increasingly individualize from their parents and try to initiate new relationships with peers and romantic partners. Given these fundamental changes in social networks and relationships, adolescents are considered to be especially prone to loneliness [6,7]. Aiming to understand who is at risk to experience feelings of loneliness, scholars have long investigated associations between personality (i.e., relatively stable patterns of thoughts, feelings, and behavior [8]) and loneliness. Previous studies indicate that, among the Big Five personality traits, lower levels of extraversion and higher levels of neuroticism are the strongest unique predictors of loneliness [9,10]. Less is known, however, about the potential differential role of each trait, possible non-linear predictive effects, and how extraversion and neuroticism interact in predicting loneliness. For example, the predictive effect of extraversion might be more pronounced for individuals with higher neuroticism [11].

To disentangle and explore these potentially complex associations, we combined polynomial regression analysis with an information-theoretic approach for model comparison [12,13]. Specifically, we derived competing theoretical hypotheses about the associations in question, translated them into corresponding statistical models, and compared their empirical evidence with information-theoretic indices that reflect the relative support of the models in the data. In addition, we explored whether the specific interplay of extraversion and neuroticism differs across loneliness facets [14].

### 1.1. Personality and Loneliness in Adolescence

Put simply, extraversion describes the tendency to socially approach others and to enjoy their company, while neuroticism characterizes an individual’s tendency to feel anxious [15,16]. Adolescents higher in extraversion may find it easier to form new friendships and experience higher levels of emotional closeness [17], be more satisfied with their social interactions [18], perceive more support from their peers [19], and be more liked and popular among their classmates [20]. In contrast, adolescents higher in neuroticism may be more insecure in their social relationships [21,22], experience lower levels of emotional closeness [17], be less satisfied with their social interactions [18], and be less liked and popular among their classmates [20]. Given these strong social implications of extraversion and neuroticism, both traits might help to identify adolescents who are at risk of developing loneliness.

Along these lines, a recent meta-analysis by Buecker et al. [9], including both cross-sectional and longitudinal studies, suggested that among the Big Five personality traits, extraversion and neuroticism are the strongest predictors of loneliness: Individuals lower in extraversion (*r* = −0.37) and higher in neuroticism (*r* = 0.36) reported higher levels of loneliness at baseline and up to 17 years later. Notably, most of the studies included in the meta-analysis used adult samples, yet similar associations were observed in studies using adolescent samples [10,19]. Age-specific analyses suggested that associations of lower extraversion with higher loneliness might be even more pronounced in adolescence than in adulthood [9,23], although these findings await replication. In sum, there is strong evidence for the predictive effects of extraversion and neuroticism for loneliness in adolescence, yet the potential differential role of each trait and their interplay are less clear.

A widely accepted conceptualization of the nature of loneliness proposed by Weiss [14] differentiates between the two facets *emotional loneliness* (i.e., the absence of an attachment figure) and *social loneliness* (i.e., the absence of a social network or the lack of belongingness). Whereas emotional and social loneliness share a common core of experiences and can thus be aggregated into an overall loneliness score [24], recent empirical work points to differential associations of the two facets with personality and to differences in developmental trajectories. For example, Buecker et al. [9] found that, on average, extraversion was more strongly associated with social loneliness and neuroticism seemed to relate to both loneliness facets equally. In a study tracking participants from a large Norwegian sample (*N* = 3116) across adolescence and young adulthood, von Soest et al. [25] found that emotional loneliness increased and social loneliness decreased over the course of seven years. Together, these studies highlight the importance of distinguishing between the two facets of loneliness [10,26].

Relatively little research has examined how personality traits relate to prospective changes in loneliness. As one of few exceptions, Mund and Neyer [27] tracked young adults across two measurement points (1995 and 2010). Accounting for the initial levels of all constructs included in their statistical model, Mund and Neyer found that, out of the Big Five, only higher neuroticism predicted subsequent 15-year increases in loneliness. In adolescence, we are aware of only one study looking at personality profiles that help distinguish trajectory classes of loneliness [23], but to the best of our knowledge, no longitudinal study exists that has examined for this age group whether and how extraversion and neuroticism are predictive of subsequent changes in loneliness. To fill this research gap, it is important to go beyond cross-sectional personality—loneliness associations and to investigate longitudinal associations while controlling for the initial level of loneliness.

### 1.2. Going beyond Linear Main Effects of Extraversion and Neuroticism

Research so far has largely focused on linear main effects in the prediction of personality traits for loneliness. This is surprising, given that as early as 1985, Eysenck and Eysenck have suggested that reactions to social stimuli of people with high neuroticism should differ significantly depending on their level of extraversion [28]. Continuing this line of argumentation, Hotard et al. [11] reasoned that neuroticism could reinforce the negative reactions individuals low in extraversion experience in social interactions, resulting in particularly strong social withdrawal of people who are both low in extraversion and high in neuroticism. Thus, whereas theory suggests more complex ramifications of extraversion and neuroticism for loneliness, to the best of our knowledge this possibility has so far not yet been tested empirically. In order to better understand how extraversion and neuroticism predict loneliness in adolescence, it is important to consider non-linear and interaction effects in addition to linear ones and to investigate both traits in an integrative manner.

**Figure 1:**
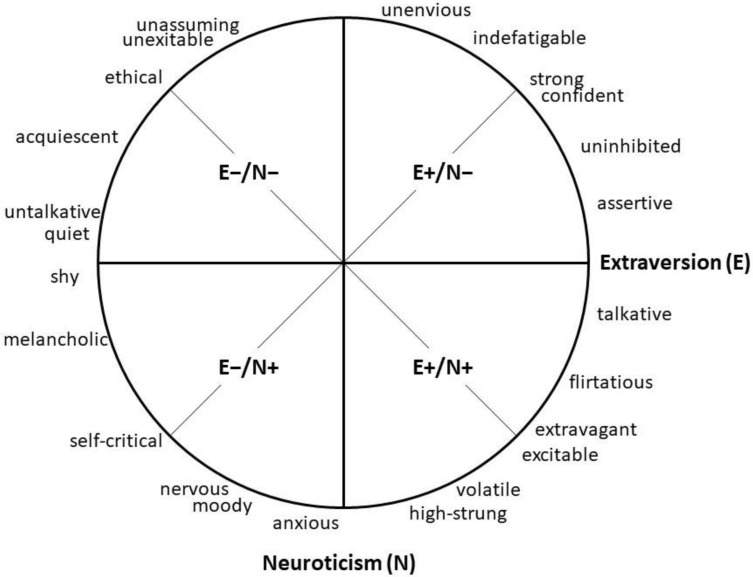
The E-N circumplex by Hofstee et al. [15]. The bold axes represent the two traits extraversion and neuroticism. In the spaces between these axes, adjectives describing different combinations of high (+) and low (−) levels of extraversion and neuroticism can be assigned. Adapted from the Journal of Personality and Social Psychology 1992, Vol. 63, No. 1, 146-163, Copyright © 1992 by the American Psychological Association. Reproduced with permission.

Unlike bipolar personality models, circumplex models take into account that descriptions of interpersonal behavior can often be assigned to more than one trait [29]. Specifically, the E-N circumplex model by Hofstee et al. [15] (see Figure 1) maps facets of extraversion and neuroticism dimensions as blends of two factors and thereby offers a framework for the integrative study of both personality traits. Spanning around two axes representing extraversion and neuroticism, the four spaces between these axes capture a range of attributes referring to the specific trait intersections. First, the combination high extraversion/low neuroticism (E+/N−) refers to individuals who are unenvious, strong, and assertive. Second, high extraversion/high neuroticism (E+/N+) describes people who are talkative, excitable, and high-strung. Third, low extraversion/low neuroticism (E−/N−) portrays quiet, acquiescent, and unassuming individuals. Fourth and finally, low extraversion/high neuroticism (E−/N+) refers to people who are anxious, self-critical, and shy. As we will illustrate in the following, both the axes and the intersections of the E-N circumplex [15] can serve as a basis to form more specific predictions for how extraversion and neuroticism, as well as their interplay, could relate to loneliness.

#### 1.2.1. Monotonous but Non-Linear Effects of Extraversion and Neuroticism

Previous studies have often estimated linear predictive effects of extraversion and neuroticism for loneliness [30,31], but the strength of these associations might vary across trait levels. Such associations can be assigned to the family of monotonic but non-linear effects and, more specifically, can take the form of saturating or exponential effects. Considering the extraversion axis in the E-N circumplex [15], one such scenario is that the beneficial effects of extraversion might saturate at higher levels: At the lower end, being very or modestly low in extraversion (e.g., very or modestly shy) might make a big difference, such that those adolescents with very low trait levels are especially prone to loneliness, whereas people who are modestly low in extraversion may not. At the higher end of extraversion, in contrast, being fairly versus very high in extraversion (e.g., fairly or very talkative) might not make much of a difference for loneliness because people at these levels of extraversion are rather unlikely to be or become lonely. Considering the neuroticism axis in the E-N circumplex [15], a second complementary scenario is that the detrimental implications of neuroticism might be exacerbated at higher levels: At the lower end, being very or modestly low in neuroticism (e.g., very or modestly unenvious) might not make much of a difference for loneliness, in the sense that people with both levels are rather unlikely to feel lonely. At the higher end of neuroticism, in contrast, being very or fairly high in neuroticism (e.g., fairly or very anxious) might make a big difference, such that individuals with very high neuroticism are at an exponential risk to experience loneliness.

In sum, monotonous but non-linear effects would indicate that the predictive effects of extraversion and/or neuroticism for loneliness either attenuate or amplify at higher trait levels. To provide a comprehensive description of both traits’ associations with loneliness, it thus appears promising to consider monotonous but non-linear associations in addition to linear ones.

#### 1.2.2. Interaction Effects between Extraversion and Neuroticism

In addition to monotonous, non-linear effects, associations might be even more complex and involve interaction effects between both traits [11,28]. Whereas empirical tests of such claims with respect to loneliness have yet to be conducted, initial support comes from research on another outcome: Interaction effects between extraversion and neuroticism have been found to predict subjective well-being beyond the main effects of each of these traits [11,32,33]. Based on these findings, we propose that the effects of extraversion and neuroticism on loneliness might moderate and mutually depend on each other, but the exact nature of this potential interplay requires further theoretical consideration.

Even though the circumplex traits are not identical to a trait by trait statistical interaction [34], the four intersections of the E-N circumplex [15] are useful to consider how different combinations of extraversion and neuroticism could relate to loneliness. The model assigns attributes that are favorable for the initiation and maintenance of social relationships to the intersection of high extraversion combined with low neuroticism (E+/N−), whereas attributes that are rather disadvantageous for people’s social lives are assigned to the intersection of low extraversion combined with high neuroticism (E−/N+). Corroborated by empirical research [9], the theoretical model suggests that lower levels of extraversion and higher levels of neuroticism are indeed associated with more loneliness. In addition, the circumplex model highlights two further intersections that are theoretically relevant but have not yet been examined empirically: higher extraversion combined with higher neuroticism and lower extraversion combined with lower neuroticism.

According to the E-N circumplex [15], individuals with higher extraversion/higher neuroticism (E+/N+) are characterized by a more extravagant and excitable nature. It is possible that such characteristics might act as a resource to get to know new people and to build a large social network. In contrast, it is also possible that these same attributes might constitute a barrier for satisfying close relationships and thus might be a source of loneliness. Similarly, two opposing predictions with regard to loneliness can be derived for individuals characterized by lower extraversion combined with lower neuroticism (E−/N−): According to the circumplex, people with this trait combination are ethical, acquiescent, and unassuming. It is possible that such attributes are less helpful for the acquisition of social contacts. In contrast, though, people with those attributes could also desire less frequent social interactions and spend less time brooding over their social relationships, and thus feel less lonely.

In a linear model, the effects of extraversion and neuroticism would simply add up, such that adolescents with both combinations (E+/N+ and E−/N−) have loneliness scores at the medium level. Alternatively, the effects of extraversion and neuroticism might mutually depend on each other, resulting in two scenarios where only specific constellations of both traits relate to increased or decreased loneliness. A first scenario is one of mutual compensation according to which the beneficial effects of higher extraversion might compensate for the detrimental effect of higher neuroticism, while the beneficial effects of lower neuroticism compensate for the detrimental effects of lower extraversion. Looking at the very low and very high ends of each trait’s spectrum, loneliness would be rather low as long as extraversion is higher or neuroticism is lower, but it would be higher when lower extraversion co-occurs with higher neuroticism. A second scenario is one of an optimal constellation according to which the beneficial effects of higher extraversion might be reinforced for those who are also lower in neuroticism but weaker for those with higher neuroticism. In turn, the detrimental effects of lower extraversion would be even stronger for people with higher neuroticism, resulting in only one specific constellation that relates to lower loneliness. Looking at the very low and very high ends of each trait’s spectrum, individuals who are either lower in extraversion or higher in neuroticism would tend to feel lonely. Only those who are both higher in extraversion and lower in neuroticism would be expected to score lower in loneliness in this scenario.

Importantly, the predictions outlined above might be less opposing when we add in a time perspective on social relationships: It is conceivable that the E+/N+ combination has in the short-term both advantages and disadvantages. In contrast, though, the combination might be detrimental in the long run in fostering existing social relationships. For example, disadvantages relating to high neuroticism might be balanced by the advantages relating to high extraversion at early relationship stages, but accelerate the development of loneliness if the relationship persists and does not develop in the expected direction, such as reaching an increase in emotional closeness. Therefore, this combination might rather be associated with loneliness changes (i.e., increases) instead of loneliness manifestations at a given point in time (i.e., cross-sectional association). Similarly, the E−/N− combination may balance harmful and helpful aspects in early relationship stages, but one could argue that low extraversion likely becomes less important once a social relationship has been established and, therefore, might be rather unrelated to changes in loneliness.

### 1.3. The Current Study

**Figure 2:**
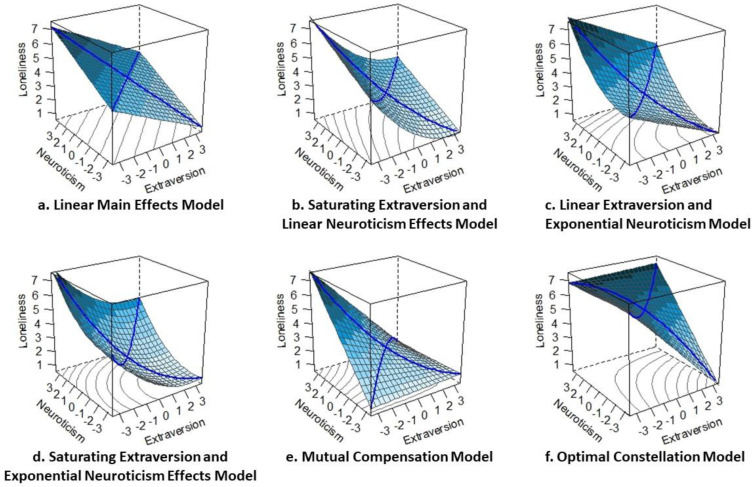
Prototypical model representations of the tested models. The bold axes represent the two traits extraversion and neuroticism. In the spaces between these axes, adjectives describing different combinations of high (+) and low (−) levels of extraversion and neuroticism can be assigned. Note. The blue color indicates the hypothesized levels of loneliness from light (low) to dark (high). Whereas model (**a**) is the only model representing mere linear main effects of extraversion and neuroticism on loneliness, models (**b**–**d**) reflect monotonous but non-linear effects of one or both traits, and models (**e**,**f**) involve linear interactions (i.e., mutual dependence) of extraversion and neuroticism.

In sum, despite a rich tradition of research on loneliness, it remains an unanswered question whether the predictive effects of extraversion and neuroticism simply add up, or whether the predictive effects are more complex and involve non-linear associations or interdependent effects between both traits. To provide a comprehensive test of possible patterns, we specified six competing hypotheses, each operationally defined with a statistical model (see Figure 2 and Table A1 for an overview). As outlined in the following, our hypotheses and models can be grouped into three categories, namely linear main effects (see Figure 2a), monotonous but non-linear effects (see Figure 2b–d), and linear interactions (mutual dependence; see Figure 2e,f).

To begin with, current empirical evidence [9] suggests linear main effects of both extraversion and neuroticism. We refer to this scenario as Linear Main Effects Hypothesis according to which lower extraversion and higher neuroticism are each associated with more loneliness (*Linear Main Effects Hypothesis*; Figure 2a). Based on theoretical accounts of associations between extraversion and neuroticism [11,28] and on theoretical implications of the E-N circumplex [15], we consider a range of potential alternative hypotheses. Importantly, these hypotheses do not necessarily contradict the Linear Main Effects Hypothesis, but rather add to its complexity.

Going beyond simple linear effects, we consider three hypotheses specifying that extraversion and/or neuroticism have monotonous but non-linear predictive effects for loneliness. First, looking at the extraversion axis within the E-N circumplex [15], the trait’s beneficial effect might saturate at higher levels *(Saturating Extraversion and Linear Neuroticism Effects Hypothesis*; Figure 2b). Second, looking at the neuroticism axis of the E-N circumplex, a complementary scenario seems possible such that the trait’s detrimental implications might be exacerbated at higher levels (*Linear Extraversion and Exponential Neuroticism Effects Hypothesis*; Figure 2c). Third, looking at the predictive effects of both traits, we might find that the main effect of extraversion saturates *and* the main effect of neuroticism increases at higher levels of the respective trait (*Saturating Extraversion and Exponential Neuroticism Effects Hypothesis*; Figure 2d).

Going beyond main effects, the third group of hypotheses considers how the effects of extraversion and neuroticism might moderate and mutually depend on each other. First, the effects of extraversion and neuroticism might compensate each other, such that only lower extraversion paired with higher neuroticism relates to higher loneliness (*Mutual Compensation Hypothesis*; Figure 2e). Second, the effects of extraversion and neuroticism might reinforce each other, such that only higher extraversion paired with lower neuroticism relates to lower loneliness (*Optimal Constellation Hypothesis*; Figure 2f).

In our study, we compared the empirical evidence for these competing hypotheses against each other by using an information-theoretic approach [12,13]. In doing so, we conducted separate analyses in which we considered cross-sectional associations versus longitudinal associations of the personality traits extraversion and neuroticisms and an overall score of loneliness. We also distinguished between emotional and social loneliness. Previous research [27] indicates that neuroticism might be particularly relevant for loneliness increases over time and that extraversion might be particularly relevant for differences in social loneliness [9,23]. Given the lack of previous research on interactive patterns between personality traits and their link with loneliness, however, we refrained from making specific predictions regarding changes in loneliness or regarding the different facets of loneliness.

## 2. Method

Hypotheses and data analyses were preregistered at https://osf.io/mx57d/ (accessed on 22 November 2021) via the Open Science Framework [35]. We analyzed data from two longitudinal studies conducted in Germany so as to compare the competing hypotheses in each of the two samples separately.

First, as Sample 1, we used data from the German Family Panel (pairfam) release 11.0 [36]. Ethical approval for the study was given by the ethics committee of the Faculty of Management, Economics and Social Sciences of the University of Cologne; a detailed description of pairfam can be found in Huinink et al. [37]. Individuals from three different birth cohorts were sampled by pairfam, born between 1971 and 1993, and additionally surveyed the children of these individuals. After age 15, these children were asked to enter the study as participants themselves and were subsequently termed *step-ups*. In this study, we only used data from adolescent participants of the step-up sample assessed at waves 10 and 11 (years of data collection: 2017–2019) who were aged between 16 and 20 at wave 10. Participants of Sample 1 were selected this way to most closely match the participants of Sample 2 with regard to the age and time of data collection. In the following, we will refer to the data collection at wave 10 as T1 and to the data collection at wave 11 as T2.

Second, as Sample 2, we used data from the SELFIE study [38], a German multimethod longitudinal study on the development of personality traits and self-esteem across major life transitions. Ethical approval for the study was given by the German Psychological Society (DGPs). SELFIE collected data from adolescents in their final year of high school and from adults awaiting their retirement at three measurement points with half-year intervals in-between. In the current study, only data from adolescent participants were used. In the following, we will refer to the data collection at the first measurement point as T1 and to the data collection at the third measurement point (i.e., the second follow-up) as T2.

### 2.1. Participants

Following the exclusion of *n* = 4 participants who did not provide loneliness ratings at T1, Sample 1 consisted of *N*_T1_ = 346 adolescents (50.00% female) aged 16–20 (*M* = 17.45, *SD* = 1.34). Most participants attended high school (45.09%), whereas the remaining ones attended schools from medium (24.28%) and lower (7.23%) school tracks within the German school system. The remaining adolescents of this sample attended other school types, such as comprehensive schools (21.10%), or provided no answer to this question. With a dropout of 18.21%, *N*_T2_ = 283 adolescents of Sample 1 also participated at T2. Compared to the continuers, the dropout group of Sample 1 was older, *t*(88.01) = 2.79, *p* = 0.006, *d* = 0.40, 95% CI [0.13, 0.68], with a small effect. With regard to gender, extraversion, neuroticism, and loneliness, no significant differences were found.

Requiring no data exclusion, Sample 2 consisted of *N*_T1_ = 237 adolescents (75.95% female) aged 15–22 (*M* = 17.73, *SD* = 1.02). Due to the inclusion criteria of SELFIE, all participants of Sample 2 attended high school (i.e., the highest school track in the German school system) at the time of data collection. With a dropout of 45.57%, *N*_T2_ = 129 adolescents of Sample 2 also participated at T2. Compared to the continuers, the dropout group of Sample 2 scored higher on extraversion *t*(230.62) = 2.40, *p* = 0.017, *d* = 0.31, 95% CI [0.05, 0.57], with a small effect. With regard to age, gender, neuroticism, and loneliness, no differences significant were found.

Since we were working with existing data, the sample size was predetermined. Nonetheless, we performed a simulation in R [39] to determine a priory power for our different samples and subsamples. Results indicated that we had a power of 99.99% or higher to obtain a significant full model when the population model explains a typical amount of variance (*R*^2^ = 0.20) in loneliness, and a power of 98.43% or higher to detect even a small effect (*R*^2^ = 0.10). The typical amount of variance was estimated on the basis of the correlations between personality traits and loneliness reported in the meta-analysis by Buecker et al. [9] and on the correlation between extraversion and neuroticism in our own data (*rs* ranging from −0.31 to −0.34; see Table 1).

### 2.2. Procedure and Measures

All measures relevant to this study were assessed via participants’ self-reports. In Sample 1, participants were interviewed once a year at home. In Sample 2, participants entered their answers into online questionnaires, either at the laboratory in Berlin or at home. In order to obtain cross-sectional and longitudinal associations, we used personality measures from T1 and loneliness measures from T1 and T2 in both samples.

#### 2.2.1. Personality Traits

In Sample 1, trait extraversion and neuroticism were measured with the short version of the German Big Five Inventory (BFI-K) [40]. Traits were assessed with four items each and participants specified their level of agreement on a Likert-type rating scale ranging from 1 (absolutely incorrect) to 5 (absolutely correct). Internal consistencies of the extraversion and neuroticism scales as indicated by Cronbach’s α were 0.72 and 0.74, respectively.

In Sample 2, trait extraversion and neuroticism were measured with the German version of the Big Five Inventory 2 (BFI-2) [41]. Specification of the level of agreement with the item content was done on a Likert-type rating scale ranging from 1 (strongly disagree) to 7 (strongly agree). Internal consistencies of the extraversion and neuroticism scales as indicated by Cronbach’s α were 0.86 and 0.88, respectively.

#### 2.2.2. Loneliness

In Sample 1, loneliness was measured with a single item translating to “I feel lonely”. Answers were given on a Likert-type rating scale ranging from 1 (not at all) to 5 (absolutely).

In Sample 2, loneliness was measured with German translations of four items from the revised University of California Los Angeles (UCLA) Loneliness Scale [42]. Specification of the level of agreement with the items’ content was done on a Likert-type rating scale ranging from 1 (strongly disagree) to 7 (strongly agree). As demonstrated by Hawkley et al. [43], the full UCLA Loneliness scale possesses a multidimensional structure representing either emotional or social aspects. Thus, in line with the suggested differentiation by Weiss [14] (for a similar approach, see [25]), this study assessed emotional loneliness with two items (“I lack companionship” and “I feel left out”) and social loneliness with two reverse-coded items (“there are people I can turn to” and “there are people I can talk to”). Based on the four items, we formed two specific loneliness scores, emotional and social loneliness, and an overall loneliness score, which is the aggregate of the former two scores. Internal consistencies of the overall loneliness, emotional loneliness, and social loneliness scales at T1 (at T2) as indicated by Cronbach’s α were 0.73 (0.78), 0.70 (0.53), and 0.86 (0.91), respectively.

### 2.3. Data Analysis

In order to test how different configurations of extraversion and neuroticism relate to loneliness, we used an information-theoretic (IT) approach for model comparison [12], following a similar strategy as described by Humberg, Dufner, et al. [13]. That is, we translated the competing hypotheses into corresponding polynomial models and compared their empirical evidence via Akaike weights, which are measures of evidence computed from the second-order Akaike information criterion (AICc) [44,45,46]. We chose the AICc for model comparison because it allowed us to compare non-nested models, avoids overfitting, and has a strong theoretical foundation in Kullback-Leibler information theory [12,44]. The global model in our analysis is the full second-order polynomial model with two predictors [47,48]:

L_n_ = b_0_ + b_1_E_n_ + b_2_N_n_ + b_3_E_n_^2^ + b_4_E_n_N_n_ + b_5_N_n_^2^ (1)

where L_n_ is the loneliness score of person n, E_n_ (extraversion) and N_n_ (neuroticism) are the predictors, E_n_N_n_ is their product, and E_n_^2^ and N_n_^2^ are their squared values. All six theoretically derived alternative hypotheses can be represented by polynomial models that are nested in the global model (Equation (1)). We defined these hypothesis-testing models by constraining the coefficients of the global model based on basic mathematical rules about quadratic equations [48]. The respective constraints are defined in Table A1. They were chosen in a way that ensures that the model’s predictions are in line with the respective hypothesis’ predictions for the entire range of realistic values of the personality variables. A detailed explanation of how the constraints were derived can be found online at https://osf.io/s8nbk/ (accessed on 22 November 2021).

#### 2.3.1. Extension of the Initial Model Set

We extended the initial model set by further models. The application of information-theoretical model comparison requires the set of models to be complete, in the sense that it contains models for all theoretically plausible (even if less expected) hypotheses [12,49]. To achieve this, we identified a number of supplementary hypotheses, which we also translated into corresponding polynomial models (see Table A1).

First, only one of the two main effects, that is, the main effect of extraversion or neuroticism, might predict loneliness when controlling for the other trait (*Linear Main Effect of Extraversion Model* and *Linear Main Effect of Neuroticism Model*). Second, these monotonously positive or negative main effects might be nonlinear, reflecting that the trait’s effect either attenuates or becomes stronger at higher levels. Overall, this adds two more models for extraversion (*Saturating Effect of Extraversion Model* and *Exponential Effect of Extraversion Model*) and neuroticism (*Exponential Effect of Neuroticism Model* and *Saturating Effect of Neuroticism Model*) each. Third, as reflected in our hypothesis set (see Figure 2b,c), it might be the case that both traits have a monotonously positive/negative main effect but that one or both of these effects are non-linear. As a supplement to the models representing our hypotheses, we added three more models. These posit monotonous main effects of both traits, where the strength of the monotonously negative effect of extraversion is stronger at higher trait levels (*Exponential Extraversion and Linear Neuroticism Effects Model*), the strength of the monotonously positive effect of neuroticism is less pronounced at higher trait levels (*Linear Extraversion and Saturating Neuroticism Effects Model*), or both (*Exponential Extraversion and Saturating Neuroticism Effects Model*). Fourth, for statistical reasons, we included an intercept-only model (*Null Model*), which represents the possibility that both extraversion and neuroticism are unrelated to loneliness, and we included the global model (Equation (1)) in which all other models are nested (*Full Model*). Combining the theoretically derived and the supplemental models, our complete model set contains a total of 19 models for competitive testing.

#### 2.3.2. Test of Competing Hypotheses

We tested all models cross-sectionally and longitudinally. For the longitudinal analyses, we used loneliness scores at T2 as the outcome variable in the global model (Equation (1)) and included loneliness at T1 as an additional predictor with a freely estimated coefficient. The nested models for the longitudinal analyses are defined by the same coefficient constraints as stated in Table A1 (e.g., the null model in the longitudinal analyses freely estimates the intercept and the coefficient of T1 loneliness). When using the overall score of loneliness as outcome, parallel, but separate analyses were conducted for each sample. Using the data of Sample 2, we re-run these analyses with emotional and social loneliness as outcome. To ensure comparable metrics across the different measures used in our two samples, we linearly transformed all scores (i.e., extraversion, neuroticism, and loneliness) of Sample 1 via the proportion of maximum scaling method (POMS) [50]. Like the variable scores from Sample 2, variable scores from Sample 1 also ranged from 1 to 7 after transformation. In addition, to facilitate interpretation, the scores of extraversion and neuroticism were centered at their respective mean within each sample.

All analyses were conducted in R [39] and R Studio [51]. In the first step of each analysis, we estimated the global model (full model) and the null model with the lm() function in the R package stats and compared their fit with a chi-square likelihood ratio test. We proceeded with the analysis only if this test revealed that the full model explained significantly more variance in the outcome than the null model. We then estimated all models in the initial model set with the sem() function in lavaan [52], using ML estimation with robust standard errors, and treating missing data with FIML. We then computed the models’ AICc values from the respective number of free parameters (K) and the maximized Log-Likelihood (LL). The AICcs were used to calculate the models’ Akaike weights with the AICcmodavg package [53]. For the computation of Akaike weights, we removed redundant models [54]. More specifically, when the difference between the maximized log-likelihood values of two nested models was less than 1 (for the rationale behind this choice, see [13]), we excluded the more complex of the two models for the computation of the Akaike weights. We tested for influential cases using the full polynomial model and three standard indicators of influence and leverage (i.e., *dfFit*, Cook’s distance D, and the *hat* value) [47,55,56], but no such cases were identified (for a more specific definition of the conditions that we applied to categorize a data point as an influential case, see the R code and preregistration provided at our OSF page at https://osf.io/4dgku/ (accessed on 22 November 2021). We used the RSA package [57] to plot the estimated regression surfaces. The code that is necessary to reproduce all results and the data of Sample 2 are provided online at our OSF page (data of Sample 1 cannot be shared publicly due to the copyright in place, but requested for scientific use from the official pairfam web page: https://www.pairfam.de/en/ (accessed on 22 November 2021).

The interpretation of the model comparisons is based on the models’ Akaike weights. The Akaike weight of a model is the likelihood that the respective model is the best model to explain the data at hand, as compared to all models in the initial model set [44,45,46]. The IT approach thereby differs from classical null-hypothesis testing in that it refrains from dichotomous decisions based on statistical significance (e.g., inspection of *p*-values). Instead, it allows for a simultaneous evaluation and comparison of the empirical evidence of all competing models and their corresponding hypotheses [12].

## 3. Results

### 3.1. Descriptive Statistics

**Figure A1:**
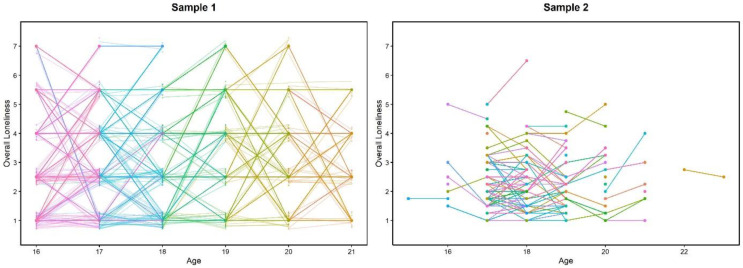
Individual changes of loneliness between T1 and T2 plotted over age. The colored dots and lines represent individual scores of overall loneliness. In Sample 1, loneliness was assessed with a one-item measure and the plot is based on data of *N* = 283 adolescents who participated at T1 and T2. To avoid overplotting and to illustrate the distribution of values within this sample, we added transparent dots and lines, which represent the true values plus noise. In Sample 2, overall loneliness was computed from the average of four items, and the plot is based on data of *N* = 129 adolescents who participated at T1 and T2. Please note that whereas loneliness is plotted over age for illustrative purposes, statistical models were estimated over measurement points of the study.

Descriptive statistics and bivariate intercorrelations at T1 and T2 can be obtained from Table 1. Compared to Sample 1, participants of Sample 2 scored higher on neuroticism, *t*(554.83) = 2.74, *p* = 0.006, *d* = 0.22, 95% CI [0.06, 0.39], and lower on overall loneliness at T1, *t*(568.33) = −3.03, *p* = 0.003, *d* = −0.23, 95% CI [−0.40, −0.07], and T2 *t*(568.33) = −4.03, *p* < 0.001, *d* = −0.36, 95% CI [−0.57, −0.14], with small effect sizes. Loneliness consistency across one year from T1 to T2 was moderate in Sample 1 (r = 0.47) and strong in Sample 2 (rs ranging from 0.63 to 0.71). On average, loneliness (and its facets) did not change across one year in both samples (see Table 1 for Cohen’s *d*). The individual differences between overall loneliness at T1 and T2 within each sample are illustrated in Figure A1. Looking at the bivariate correlations between personality traits and loneliness in both samples, all variables were significantly related to each other except (a) neuroticism and social loneliness and (b) neuroticism and overall loneliness at T2 in Sample 2. Looking at the role of demographic characteristics, older age was associated with higher neuroticism (*r* = 0.12, *p* = 0.023) and overall loneliness at T1 (*r* = 0.16, *p* = 0.003) in Sample 1, and female participants reported higher neuroticism in both samples (Sample 1: *r* = 0.33, *p* < 0.001; Sample 2: *r* = 0.38, *p* < 0.001). Age and gender were not significantly related to any of the other study variables.

### 3.2. Predicting Loneliness from the Interplay of Extraversion and Neuroticism: Model Comparison Analyses

For each of our analyses, Table 2 presents the 95% *confidence set* of models [12] of all analyses: Starting with the model with the largest Akaike weight and continuing with the next best models, models were added to the confidence set until the cumulated weights in the set exceeded 95%. This way, the confidence set includes the models with the highest likelihood of being the best models among all models in the initial set. Models which are not included in this set have very little empirical support (i.e., a likelihood <5% of being the best model to explain the data) and were therefore excluded from interpretation. In the case that the global model was included in the confidence set, we interpreted its coefficients by use of response surface methodology, following the guidance by Edwards [47] and Humberg, Nestler, et al. [58]. This allowed us to detect any empirical patterns that are not represented by the models of our hypothesis set. In the following, we will explain the results of each model in the confidence set in detail. This will be done first for the cross-sectional models and then for the longitudinal models.

#### 3.2.1. Cross-Sectional Analyses

In this section, we present the results of the cross-sectional models in several steps: First, starting with the models predicting overall loneliness, we will outline the results from Sample 1 and 2 one after each other. Second, we report on the results from the models predicting the loneliness facets based on the data of Sample 2. Then, as a final step, we will summarize the most relevant findings of the cross-sectional models across samples and loneliness scales.

##### Overall Loneliness

**Figure 3:**
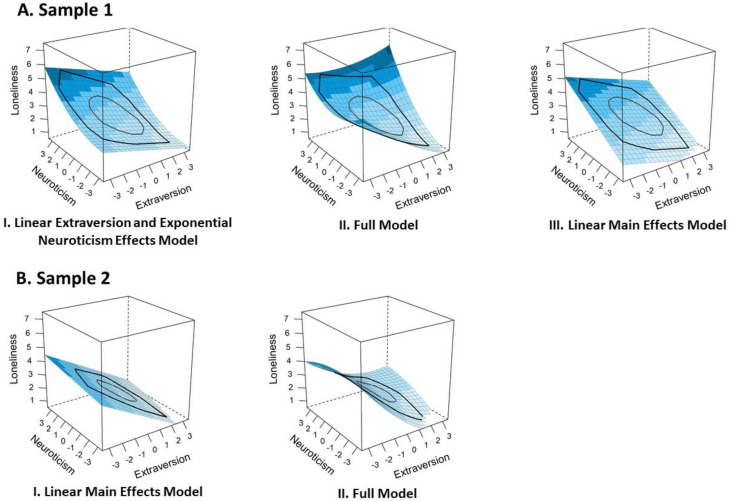
Graphs of the models in the confidence set: cross-sectional models predicting overall loneliness in Sample 1 (Panel **A**) and Sample 2 (Panel **B**). The blue color indicates the levels of loneliness from light (low) to dark (high). The black lines represent the bagplot that indicates the distribution of extraversion and neuroticism. The interpretation of the surface must be restricted to this area.

In Sample 1, three models predicting overall loneliness at T1 were included in the confidence set (see Figure 3 Panel A). Together, the three models provided evidence for the main effects of both personality traits, for non-linear effects, and for the possibility of a weak interaction effect. First, the Linear Extraversion and Exponential Neuroticism Effects Model had a likelihood of 42% (*w* = 0.42) of being the best model out of our alternative models. It showed a stronger association between neuroticism and loneliness as compared to the association between extraversion and loneliness. Going beyond the linear negative/positive associations between extraversion/neuroticism and loneliness, this model indicated a slightly curvilinear nature of the effect of neuroticism. That is, adolescents with higher neuroticism felt lonelier in general, and this effect was even more pronounced the higher the level of neuroticism was (i.e., exponential effect).

Second, the Full Model was the next best model (*w* = 0.33). In addition to the previously described effects, the Full Model reflected the possibility that the negative association of extraversion and loneliness might be weaker at higher levels of the trait (i.e., saturating effect) and the possibility of a weak positive interaction between extraversion and neuroticism. That is, the effects of neuroticism might be slightly more pronounced for adolescents with higher levels of extraversion. Conversely, the effects of extraversion might be slightly more pronounced for adolescents with higher levels of neuroticism.

Finally, the set was completed by the Linear Main Effects Model (*w* = 0.25). According to the Linear Main Effects Model, higher loneliness in adolescence is simply related to lower extraversion and higher neuroticism without any curvilinear or interaction effects.

In Sample 2, the confidence set included two models (see Figure 3 Panel B), which provided support for the main effects of both personality traits and weak evidence for non-linear effects and a positive interaction effect. The Linear Main Effects Model had the most evidence (*w* = 0.66) out of the models predicting overall loneliness at T1. Thus, in this sample, adolescents’ loneliness was linearly predicted from lower extraversion and higher neuroticism, with a stronger association between extraversion and loneliness compared to the association between neuroticism and loneliness. The confidence set also included the Full Model (*w* = 0.29), which pointed to the possibility of three additional effects. As in Sample 1, the Full Model indicated that the negative effect of extraversion might be saturating (i.e., weaker association between extraversion and loneliness at higher levels of extraversion) and that extraversion and neuroticism might positively interact when predicting loneliness. Furthermore, deviating from the results in Sample 1, the Full Model in Sample 2 reflected the possibility that the positive effect of neuroticism might also be saturating in this sample. Given that this effect was very small, however, it should be considered with caution.

##### Emotional and Social Loneliness

**Figure 4:**
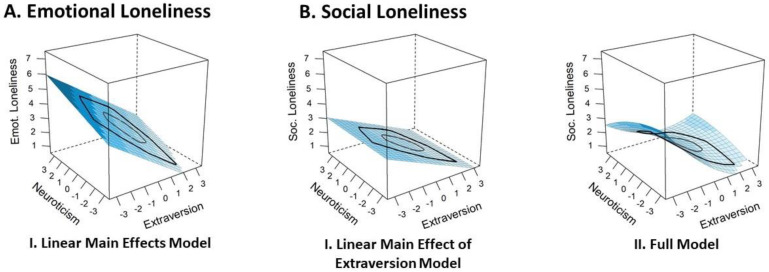
Graphs of the models in the confidence set: cross-sectional models predicting emotional loneliness (Panel **A**) and social loneliness (Panel **B**). Plots are based on the data of Sample 2 only. The blue color indicates the levels of loneliness from light (low) to dark (high). The black lines represent the bagplot that indicates the distribution of extraversion and neuroticism. The interpretation of the surface must be restricted to this area.

Being available in Sample 2 only, we differentiated between the specific loneliness facets. In the case of emotional loneliness, the confidence set included only the Linear Main Effects Model (see Figure 4 Panel A), which had a 100% likelihood (*w* = 1) of being the best model predicting emotional loneliness. Both traits linearly predicted adolescents’ emotional loneliness and the effect of extraversion exceeded the effect of neuroticism. Thus, as for overall loneliness in Sample 2, this model provided evidence for the main effects of both personality traits, but not for non-linear effects or interactions in the case of emotional loneliness.

In the case of social loneliness, the confidence set included two models (see Figure 4 Panel B). Together, the two models provided strong evidence for the main effect of extraversion along with the possibility of non-linear effects of both traits and of a weak interaction. First, the Linear Main Effect of Extraversion Model had a likelihood of 84% (*w* = 0.84) of being the best model in the model set, indicating that higher social loneliness was linearly related to adolescents’ lower extraversion only. Second, the Full Model (*w* = 0.16) diversified the picture in three ways: (1) by the possibility that the strong negative effect of extraversion on social loneliness was less pronounced at higher trait levels (i.e., saturating effect), (2) by the possibility of a weak inverse u-shaped effect of neuroticism (i.e., an overall positive but saturating effect of neuroticism on social loneliness for 89.03% of the participants, but a negative effect on social loneliness for the remaining 10.97% at the highest end of the neuroticism spectrum), and (3) by the possibility of a positive interaction between extraversion and neuroticism (i.e., the effects of both traits reinforce each other). It should be noted, however, that all effects except the negative linear main effect of extraversion were very small and should not be overinterpreted. In addition, comparing the two models predicting social loneliness, the Linear Main Effect of Extraversion Model was 5.25 times more likely than the Full Model (*evidence ratio* 0.84/0.16 = 5.25), so the nuanced nature of the effects as reflected by the Full Model should be considered with caution.

##### Summary of Cross-Sectional Results

An integrative view on the commonalities of the cross-sectional models across both samples implies strong evidence for the notion that both lower extraversion and higher neuroticism are related to adolescents’ higher overall loneliness. Whereas neuroticism was the strongest predictor of overall loneliness in Sample 1, extraversion appeared as the more predictive personality trait in Sample 2. Going beyond linear main effects, results provided weak but consistent support for a saturating effect of extraversion and a positive interaction effect of extraversion and neuroticism on loneliness. Moreover, the results of Sample 1 provided strong support for an exponential effect of neuroticism, but this was not replicated in Sample 2. Facet-specific analyses in Sample 2 pointed to distinct effects of extraversion and neuroticism with regard to emotional versus social loneliness: Whereas both lower extraversion and higher neuroticism appeared to represent relevant correlates of adolescents’ higher emotional loneliness, results supported a linear main effect of extraversion only in the case of social loneliness, which might be saturating at higher levels. Thus, adolescents’ social loneliness seemed to relate exclusively or at least predominantly to their extraversion and less to their levels of neuroticism.

#### 3.2.2. Longitudinal Analyses

**Figure 5:**
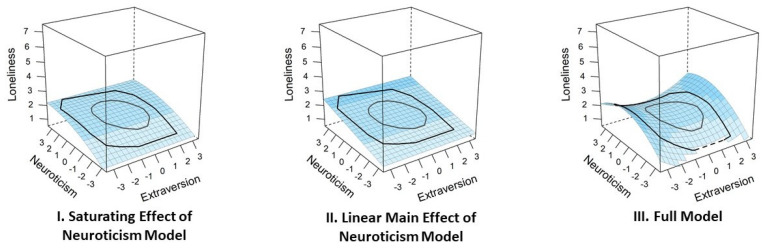
Graphs of the models in the confidence set: models predicting overall loneliness change. Plots are based on the data of Sample 1 only. The blue color indicates the levels of loneliness increases from light (very weak) to medium (weak). The black lines represent the bagplot that indicates the distribution of extraversion and neuroticism. The interpretation of the surface must be restricted to this area.

In Sample 1, three models predicting overall loneliness at T2 while controlling for loneliness at T1 (i.e., predicting relative change in loneliness) were included in the confidence set (see Figure 5). Together, the three models provided strong evidence for a (non-linear) main effect of neuroticism, but rather little evidence for an effect of extraversion or for an interaction effect. First, the saturating effect of the neuroticism model had a likelihood of 50% (*w* = 0.50) of being the best model out of the alternatives. Accordingly, adolescents with higher neuroticism were generally at higher risk to increase in loneliness and the strength of this effect differed across the trait levels: at the lower end, being very low or modestly low in neuroticism appeared to make a big difference for increases in loneliness. At the higher end of neuroticism, however, loneliness of both adolescents with fairly high versus very high neuroticism is likely to increase, but the exact level of neuroticism might not make much of a difference for the amount of this increase. Extraversion, by contrast, did not seem to predict loneliness changes at all.

Second, the Linear Main Effect of Neuroticism Model was the next best model (*w* = 0.31) Like the first model in the confidence set, this model indicated that adolescents with higher neuroticism were more likely to increase in loneliness from T1 to T2 and that extraversion played no role in this. Contrary to the first model, however, the second model did not indicate that longitudinal associations differ across different levels of neuroticism. Finally, the Full Model (*w* = 0.19) reflected the additional possibility of a negative saturating effect of extraversion and of a positive interaction between extraversion and neuroticism. However, all additional effects were rather small.

In Sample 2, extraversion and neuroticism did not explain any additional variance of loneliness at T2 when controlling for loneliness at T1. This was the case for both overall loneliness as well as the two facets. Therefore, we did not proceed with longitudinal model comparisons in Sample 2. Altogether, whereas it is important to note that analyses were restricted to Sample 1, the longitudinal models provided support for a positive effect of neuroticism on loneliness increases across one year, which might be saturating. In contrast, they provided no evidence for any longitudinal effects of extraversion on loneliness changes.

## 4. Discussion

The goal of this study was to examine the interplay of extraversion and neuroticism in predicting loneliness and its facets in late adolescence. To this aim, we specified six competing hypotheses based on theory and previous research and tested them competitively in two samples of almost 600 late adolescents in total. We obtained four sets of major findings: First, at the cross-sectional level, there was evidence for more complex, non-linear effects in addition to the previously established linear effects of extraversion and neuroticism on loneliness, but only little evidence for interaction effects. Second, specific associative patterns differed between emotional and social loneliness facets. Third, longitudinal changes in loneliness were mainly related to neuroticism. Finally, as a methodological aspect, inconsistencies across our two samples pointed to the relevance of the studied population. In the following, we will discuss these aspects in more detail, refer to implications for theory and adolescent development, and outline directions for future research.

### 4.1. The Distinct Roles of Extraversion and Neuroticism

To elucidate the relative importance of extraversion and neuroticism for adolescents’ loneliness, we compared the effects of these two traits (1) at the cross-sectional level, (2) across loneliness facets, and (3) longitudinally. As outlined in the following, our findings imply that the distinct roles of both personality traits for loneliness might vary depending on which of these three perspectives is taken.

In line with previous research [9], both lower extraversion and higher neuroticism characterized adolescents who felt more lonely as compared to others. Looking at the relative importance of each trait for loneliness, neuroticism was the strongest predictor in Sample 1, but extraversion was the strongest predictor in Sample 2. This difference might originate in the fact that the two samples varied in three important ways. First, in Sample 1 we assessed loneliness with a direct item that required labeling oneself as (more or less) lonely, whereas Sample 2 involved a more indirect measure (i.e., items of the UCLA loneliness scale [42]) which avoids the term *loneliness* [59]. Even though both types of measures have shown high convergent validity [59], a self-concept that involves labeling oneself as lonely might be more closely related to neuroticism at least in adolescence and thus, could explain the stronger association between the trait and the direct measure of loneliness. Second, the loneliness items used in Sample 2 [42] refer more to social content than those in Sample 1. Despite reflecting different aspects of loneliness, both emotional and social loneliness refer to a person’s social relationships (i.e., an attachment figure and a social network [14]) and might therefore explain the stronger association with extraversion. Third, Sample 1 was more diverse with respect to gender and educational background than Sample 2. Given that the function of personality traits can differ for boys and girls [60] and across school tracks [61], extraversion and neuroticism might have played different roles for adolescents’ loneliness based on the samples’ demographic compositions.

Our results further indicated different cross-sectional patterns across loneliness facets. Whereas both extraversion and neuroticism were associated with emotional loneliness, only extraversion was associated with social loneliness. This finding is largely consistent with previous research [9] and suggests that different behaviors and cognitions might be associated with emotional and social loneliness. Specifically, our results highlight the role of extraversion for feeling embedded into a social network: adolescents with higher extraversion enjoy being around others, are talkative, and initiate or approach social interactions [15,16], and therefore might find it easier to build a functioning social network. Importantly, previous research indicated that the quantity of social contacts is only weakly associated with loneliness [1]. Instead, based on the subjective nature of loneliness [62], adolescents’ perceived quality of social contacts and their self-identification as someone with good social relationships appears to be more crucial. In the future, studies should aim to identify the processes that are involved in the interplay between personality traits and the two facets of emotional and social loneliness to provide specific support to those adolescents who are more prone to suffer from one or both loneliness types (for an overview on existing loneliness intervention strategies, see [63]).

We found that longitudinal changes in loneliness mainly related to neuroticism, whereas extraversion seemed to play only a minor role. In line with Mund and Neyer’s [27] study tracking young adults, our results indicated that adolescents with higher neuroticism were more likely to show an increase in loneliness within the following year. Thus, acting and feeling in a more anxious and nervous manner, as it is typical for individuals with higher neuroticism [15,16], might be detrimental to the development of social relationships in the long run, for example by fostering insecurity [21] and undermining emotional closeness [17]. The role of extraversion, by contrast, might be primarily related to adolescents’ momentary experience of their social relationships and loneliness.

In sum, our results suggest that, at the cross-sectional level, both extraversion and neuroticism might be important predictors of loneliness in adolescence. A more differentiated picture appears, however, when looking at facet-specific effects: both traits were associated with adolescents’ emotional loneliness, but only extraversion was linked to social loneliness. Neuroticism, in turn, appears to be particularly relevant to loneliness changes, as suggested by our longitudinal results. Given these differences across loneliness facets and cross-sectional versus longitudinal results, future studies investigating associations between personality traits and loneliness should carefully choose the ways in which loneliness is conceptualized and the time-window during which it is observed.

### 4.2. There Is More: Tentative Support for Non-Linear and Interaction Effects

Across both samples and across most loneliness facets, results provided consistent support for linear main effects of extraversion and neuroticism. Specifically, findings mainly supported the Linear Main Effects Hypothesis (cross-sectional analyses) and the Linear Main Effect of Neuroticism Hypothesis (longitudinal analyses). Extending this picture, we also found tentative evidence for a non-linear nature of all of these main effects. In addition, there was some, albeit weak, support for the possibility that the effects of extraversion and neuroticism might mutually depend on each other (i.e., interaction effects).

#### 4.2.1. Stronger Effects at One End of the Scale: Saturating Extraversion and Exponential Neuroticism Effects

The cross-sectional analyses indicated a saturating effect of extraversion (as indicated by the Full Model), such that its negative association with loneliness was weaker for adolescents with very high extraversion scores. To illustrate this effect with the E-N circumplex [15], being fairly or very high in extraversion, which relates to characteristics of being talkative or assertive, might not make much of a difference for adolescents’ loneliness. In contrast, adolescents who are very, rather than only modestly, shy, quiet, and untalkative (i.e., at the low end of extraversion) during social interactions might be much more likely to feel lonely. With respect to neuroticism, our results indicated a non-linear effect in the opposite direction: The effect of neuroticism appeared to be exponential, suggesting that the association between neuroticism and loneliness was stronger for adolescents with very high neuroticism scores. Again, illustrating this effect with the E-N circumplex [15], adolescents who are very (rather than only fairly) anxious, moody, and high-strung (i.e., at the high end of neuroticism) during social interactions might be especially prone to feeling lonely. In contrast, being modestly or low in neuroticism, which relates to characteristics of being quiet or unenvious, might not make much of a difference for feelings of loneliness.

Altogether, our results provide support for the Saturating Extraversion and Exponential Neuroticism effects hypothesis at the cross-sectional level. This suggests that extraversion and neuroticism might not serve as continuous protection or vulnerability factors for loneliness in adolescence. In the case of extraversion, especially those adolescents with very low trait levels might require help, whereas being at the mid-range might be a sufficient resource against loneliness. Conversely, adolescents with very high neuroticism might be considered as a high-risk group for loneliness, whereas being at the mid-range might reduce this risk dramatically. Given these non-linear associations of extraversion and neuroticism with loneliness, future research and intervention programs should focus on certain areas of each trait’s range.

Of note, evidence for these more complex patterns was not conclusive. In the case of extraversion, saturating effects were found to be consistent across samples. At the same time, effects were rather small. In the case of neuroticism, evidence for an exponential effect was relatively strong in Sample 1, but could not be replicated in Sample 2. Instead, findings in Sample 2 even pointed to the possibility that the positive effect of neuroticism on loneliness was saturating, although evidence for this finding was only weak. Again, differences across our two samples might explain these divergent findings. First, inconsistencies might origin in the use of different measures and differences in the samples’ demographic compositions. For example, facet-specific analyses in Sample 2 showed that the evidence for a saturating effect of neuroticism was only given for social, but not for emotional loneliness. Accordingly, the way loneliness is conceptualized and measured appears to make a difference. Second, adolescents of Sample 2 scored lower on loneliness than those of Sample 1. Therefore, bottom effects might have disguised an exponential effect of neuroticism on loneliness in Sample 2. Overall, the results involving non-linear effects have to be regarded with caution and further replication attempts should be initiated. In addition, future studies should explore the potential moderating role of used loneliness measures and sample characteristics.

Turning to the longitudinal analyses, the results for Sample 1 extend previous research [27] by indicating that the positive effect of neuroticism on loneliness changes might be saturating. This result contrasts the detected cross-sectional associations, which indicated an exponential risk to experience loneliness for adolescents with higher neuroticism. Thus, higher neuroticism predicted loneliness increases in general, but there was no large difference between adolescents who scored high vs. very high on neuroticism. Possibly, there is a ceiling effect, such that the degree to which loneliness can increase within one year is limited. As an alternative explanation, it should be considered that neuroticism is also recognized as a source of vulnerability in clinical contexts [64]: Next to feeling lonely, individuals with very high neuroticism are likely to experience other forms of psychological distress [65,66,67]. Therefore, adolescents who score very high on neuroticism might get help and therefore develop in a similar way as their peers with slightly lower trait scores. Notably, given that we could test for longitudinal associations in one sample only, our findings on neuroticism and loneliness changes need to be replicated in future studies.

#### 4.2.2. Mutual Dependence: Positive Interactions between Extraversion and Neuroticism

Providing some—albeit weak—support for our predictions based on personality theory [11,15,28], we found initial evidence for interaction effects between extraversion and neuroticism in the prediction of loneliness at the cross-sectional and longitudinal level (as indicated by the corresponding Full Models). Specifically, there was no support for the Mutual Compensation Hypothesis. Neither could higher extraversion buffer the effect of higher neuroticism nor could lower neuroticism buffer the effect of lower extraversion beyond their additive effects. There was, however, some support for the Optimal Constellation Hypothesis in the case of overall loneliness and social loneliness, indicating that the effects of extraversion and neuroticism might reinforce each other. Illustrating this with the E-N circumplex [15], this would mean that only adolescents who are at the high end of extraversion and at the low end of neuroticism, which relates to acting strong, confident, and indefatigable during social interactions, might have a reduced likelihood to feel lonely. In this combination, the beneficial effects of higher extraversion and lower neuroticism might even go beyond the additive effects of both traits. In contrast, adolescents with all other combinations (i.e., adolescents with either lower extraversion or higher neuroticism) would have a relatively high risk to feel lonely. Moreover, this risk might be even multiplied for those who tend to act self-critical, nervous, and moody (i.e., who are at the low end of extraversion and at the high end of neuroticism [15]). Thus, while the combination of higher extraversion and lower neuroticism may be a protective factor against loneliness, the individual traits may not be. As such, it might be prudent to pay particular attention to adolescents who do not possess this combination of trait levels, as they may be at higher risk of feeling lonely.

Altogether, our findings provide the first evidence for the existence of positive interaction effects between extraversion and neuroticism. Whereas most effects were only weak and results need to be replicated in both adolescent and non-adolescent samples, the effects of extraversion and neuroticism might not simply add up to a certain loneliness level, but reinforce each other instead. Future research should therefore move beyond the exclusive consideration of linear effects: Although our findings should be considered preliminary, they clearly highlight the importance of considering more complex effects (i.e., non-linear and interaction effects) of personality traits on loneliness in theoretical and statistical models for a more precise understanding of this interplay [68].

### 4.3. Implications for Adolescent Development

In addition to our findings on the importance of extraversion and neuroticism for loneliness, our results might have a number of implications for adolescent development. First, loneliness and its facets were relatively stable across one year. Specifically, the stability of loneliness was moderate in Sample 1 and strong in Sample 2 and thus comparable to the stabilities reported by Vanhalst et al. [23] (*rs* ranging between 0.40 and 0.66), who measured loneliness of adolescents across a five-year period (ages 15–20). Accordingly, our findings add further empirical evidence to the definition of loneliness as a trait-like construct [59,69].

Second, loneliness did not change at the mean level across one year. This finding is in line with previous longitudinal research [59]. Nevertheless, it is noteworthy to mention that most participants in Sample 2 transitioned out of high school between the two measurement points. Although this transition has been related to fundamental changes across different characteristics [17,70,71,72], the loneliness of these adolescents did not increase (or decrease) on average. There are at least three explanations for this finding. First, most adolescents in Sample 2 might have had sufficient resources to cope with this important transition. Second, it could be the case that loneliness at this age is only weakly related to environmental as opposed to heritable factors [69]. Finally, the time window of the study might have been too small to detect any transition-related changes in loneliness. Whereas it is important to note that these temporal dynamics are not well understood yet and are likely to vary across individuals [73], most adolescents might remain in their familiar social environments when finishing school first, and start to individuate from their parents and contacts from school [5] with some temporal delay. Thus, loneliness changes in this context might not occur right after graduation, but later. To test this possibility, longitudinal studies tracking loneliness of adolescents across their transition out of school and thereafter for a longer time are required.

Third, our findings suggest that adolescents’ risk to experience loneliness should normatively decrease as they grow older because, on average, neuroticism decreases on the way from adolescence to young adulthood [8]. This is consistent with both theory on the co-development of a mature personality and the successful adaption to new social roles [74] and empirical findings on the life-span development of loneliness [59,75]. Moreover, our findings imply that personality maturation might primarily reduce the risk to experience emotional loneliness because only this loneliness facet was associated with neuroticism in our data.

### 4.4. Limitations

A number of limitations should be kept in mind when interpreting our results. First, our results were solely based on self-reports. Whereas the subjective experience is a core feature of loneliness [62] and other ratings of loneliness should be treated with caution when exclusively used [76], our results might be partly based on shared measurement variance [77]. Therefore, it might be helpful to use other reports of either personality traits or loneliness. Along these lines, Matthews et al. [78] analyzed data of 18-year olds from a large representative sample from the UK (*N* = 2232) and found that the personality-loneliness associations could be established for other-rated extraversion and neuroticism, too. Therefore, we expect that similar patterns as those indicated by our findings would be observed when using other-rated personality traits to predict loneliness.

Second, our longitudinal analyses were based on a relatively short time interval of one year. Therefore, our results cannot inform on how (the interplay of) extraversion and neuroticism may predict adolescents’ loneliness changes over the course of several years. In the case of Sample 2, the stability of loneliness was very high and personality did not explain any additional variance after controlling for loneliness at T1. Future studies on individual differences in loneliness should investigate longitudinal effects over the course of several years while at the same time covering not only overall loneliness but additionally differentiating between loneliness facets.

Third, our two samples had different strengths and limitations. Specifically, Sample 1 provided the opportunity to study associations between personality traits and loneliness in a sample that was relatively large and diverse with regard to education and gender, but the BFI-K [40] that was used to assess personality traits represents a relatively short scale and loneliness was assessed with one item only. Whereas both of these measures are economic and widely accepted, scales including more items might be more reliable and provide a more nuanced picture [79]. In Sample 2, more differentiated measures of both personality traits (i.e., BFI-2 [16]) and loneliness (i.e., four UCLA loneliness scale items [42]) were used. Whereas these measures provided a broader operationalization of both constructs and the possibility to distinguish between loneliness facets [14], Sample 2 was smaller and overrepresented female and highly educated adolescents. Therefore, it remains to be clarified whether the results generalize to male adolescents or those from lower school tracks. In addition, using the full UCLA loneliness scale [42] instead of four items might lead to more reliable results. By running parallel analyses with two samples, we aimed to account for the limitation of each sample and to provide a broader picture. In future research, however, it would be even better to use data that are based on both large and diverse samples and on differentiated measures.

## 5. Conclusions

Our study is the first to examine non-linear and interaction effects of extraversion and neuroticism on loneliness and the first to explore these associations in longitudinal data in adolescence. At the cross-sectional level, we found strong evidence for linear main effects of both traits, with additional hints that the negative effect of extraversion might saturate and that the positive effect of neuroticism might be exponential. Importantly, both personality traits were associated with overall loneliness and emotional loneliness, but only extraversion was related to social loneliness. Longitudinally, our findings suggested that only neuroticism predicted loneliness changes and provided tentative evidence for a saturating nature of this effect. Finally, there was some (albeit weak) evidence for positive interaction effects between extraversion and neuroticism. Our results contribute to a more nuanced and integrative understanding of the way personality relates to loneliness in adolescence. They also emphasize the importance of differentiating between emotional and social aspects of loneliness. To conclude, we hope that our study inspires future research to investigate the interplay between personality traits and loneliness in a more nuanced and integrative manner. As a next step, we propose that studies with large and diverse samples, tracking adolescents’ personality traits and loneliness across several years and measurement points are required in order to shed further light on these more complex associations.

## Figures and Tables

**Table 1:** Descriptive statistics, internal consistencies, and intercorrelations.

								Intercorrelations							
								T1 Variables					T2 Variables		
	*M*_T1_	*SD*_T1_	*M*_T2_	*SD*_T2_	*d*	α_T1_	α_T2_	E	N	L	L(e)	L(s)	L	L(e)	L(s)
**Sample 1**															
E	4.54	1.22				0.72							**−0.21**		
N	3.60	1.26				0.74		**−0.34**					**0.34**		
L	2.52	1.61	2.71	1.64	0.09	--		**−0.32**	**0.45**				**0.47**		
**Sample 2**															
E	4.69	0.95				0.86							**−0.44**	**−0.40**	**−0.38**
N	3.87	1.07				0.88		**−0.31**					0.15	**0.19**	0.07
L	2.20	0.94	2.19	0.96	0.00	0.73	0.78	**−0.57**	**0.31**				**0.71**		
L(e)	2.72	1.30	2.69	1.16	−0.06	0.70	0.53	**−0.54**	**0.39**	**0.89**			0.67	**0.63**	
L(s)	1.68	0.93	1.70	1.01	0.08	0.86	0.91	**−0.41**	0.09	**0.78**	**0.41**		0.57	0.40	**0.63**

Note: E = extraversion, N = neuroticism, L = loneliness, L(e) = emotional loneliness, L(s) = social loneliness. Results are based on *N*_T1_ = 346 and *N*_T2_ = 283 observations in Sample 1 and *N*_T1_ = 237 and *N*_T2_ = 129 observations in Sample 2. For reasons of comparability across samples, we transformed all variables of Sample 1 via POMS prior to the analysis. Internal consistencies are provided as Cronbach’s alpha (α). Bivariate correlations in bold font were significant at *p* < 0.05. Underlined intercorrelations represent retest reliabilities (*r*_T1,T2_).

**Table 2:** 95% confidence sets of models predicting loneliness from extraversion and neuroticism.

	*b*_1_	*b*_2_	*b*_3_	*b*_4_	*b*_5_	*K*	LL	AIC_c_	*w*	Adj. *R*^2^_Model_	Adj. *R*^2^_Full Model_
Cross-sectional											
**Overall loneliness**											
*Sample 1*											0.237
Linear Extraversion and Exponential Neuroticism Model	−0.26	0.47	0	0	0.07	5	−608.42	1227.02	0.42	0.233	
Full Model	−0.26	0.47	0.04	0.11	0.11	7	−606.59	1227.51	0.33	0.237	
Linear Main Effects Model	−0.25	0.49	0	0	0	4	−609.98	1228.08	0.25	0.229	
*Sample 2*											0.347
Linear Main Effects Model	−0.52	0.13	0	0	0	4	−271.22	550.61	0.66	0.342	
Full Model	−0.51	0.13	0.07	0.07	−0.04	7	−268.88	552.25	0.29	0.347	
**Emotional loneliness**											0.335
Linear Main Effects Model	−0.64	0.30	0	0	0	4	−348.43	705.03	1	0.338	
**Social loneliness**											0.165
Linear Main Effect of Extraversion Model	−0.40	0	0	0	0	3	−296.95	600.00	0.84	0.161	
Full Model	−0.39	−0.03	0.08	0.08	−0.03	7	−294.44	603.36	0.16	0.165	
**Longitudinal**											
**Overall loneliness (change)**											0.254
Saturating Effect of Neuroticism Model	0	0.26	0	0	−0.04	5	−499.99	1010.15	0.50	0.251	
Linear Main Effect of Neuroticism Model	0	0.23	0	0	0	4	−501.51	1011.14	0.31	0.246	
Full Model	−0.04	0.25	0.08	0.04	−0.10	8	−497.86	1012.14	0.19	0.254	

Note: Results are based on *N*_T1_ = 346 and *N*_T2_ = 283 observations in Sample 1 and *N*_T1_ = 237 and *N*_T2_ = 129 observations in Sample 2. Following an information-theoretic approach for model comparison, we do not report *p*-values. Instead, interpretation should be based on the models’ Akaike weights that reflect the relative evidence for all competing models. *K* = number of estimated parameters; LL = maximized Log-Likelihood; AIC_c_ = second-order Akaike information criterion; *w* = Akaike weight of the model (i.e., likelihood of being the best model in the 95% confidence set); adj. *R*^2^ = adjusted *R*^2^; *b*_1_ to *b*_5_ refer to regression coefficients of the full polynomial model L_n_ = b_0_ + b_1_E_n_ + b_2_N_n_ + b_3_E_n_^2^ + b_4_E_n_N_n_ + b_5_N_n_^2^. For reasons of comparability across samples, we transformed all variables of Sample 1 via POMS prior to the analysis. Results for emotional and social loneliness are based on the data of Sample 2 only. Longitudinal analyses additionally controlled for loneliness at T1. In Sample 2, we could not compute longitudinal results because the predictors explained no variance after controlling for loneliness at T1.

## Data Availability

Data of Sample 1 cannot be shared publicly due to the copyright in place, but requested for scientific use from the official pairfam web page (https://www.pairfam.de/en/ (accessed on 22 November 2021). Data of Sample 2 are available at https://osf.io/4dgku/ (accessed on 22 November 2021).

## Appendix A

**Table A1:** Hypotheses and corresponding polynomial regression models.

Nr.	Hypothesis	Model Name: **Corresponding Model Constraints Imposed on the Coefficients of the Full Second−Order Polynomial Model** L_n_ = b_0_ + b_1_E_n_ + b_2_N_n_ + b_3_E_n_^2^ + b_4_E_n_N_n_ + b_5_N_n_^2^	**Figure**
1		Null Model: b_1_= b_2_ = b_3_ = b_4_ = b_5_ = 0	
2		Full Model: No constraints	
*Linear main effects*			
**3**	**Lower extraversion and higher neuroticism are linearly related to higher loneliness**	**Linear Main Effects Model:** **b_1_ < 0, b_2_ > 0,** **b_3_ = b_4_ = b_5_ = 0**	**2a**
4	Lower extraversion is linearly related to higher loneliness; no effect of neuroticism	Linear Main Effect of Extraversion Model: b_1_ < 0, b_2_ = b_3_ = b_4_ = b_5_ = 0	
5	Higher neuroticism is linearly related to higher loneliness; no effect of extraversion	Linear Main Effect of Neuroticism Model: b_2_ > 0, b_1_ = b_3_ = b_4_ = b_5_ = 0	
*Monotonous but non−linear models*			
6	Higher extraversion relates to lower loneliness and this saturates; no effect of neuroticism	Saturating Effect of Extraversion Model: b_3_ > 0, −b_1_ > 2b_3_E_max_, b_2_ = b_4_ = b_5_ = 0	
7	Higher extraversion relates to lower loneliness and this effect is exponential; no effect of neuroticism	Exponential Effect of Extraversion Model: b_3_ < 0, −b_1_ > 2b_3_E_min_, b_2_ = b_4_ = b_5_ = 0	
8	Higher neuroticism relates to higher loneliness and this effect is exponential; no effect of extraversion	Exponential Effect of Neuroticism Model: b_5_ > 0, −b_2_ < 2b_5_N_min_, b_1_ = b_3_ = b_4_ = 0	
9	Higher neuroticism relates to higher loneliness and this effect saturates; no effect of extraversion	Saturating Effect of Neuroticism Model: b_5_ < 0, −b_2_ < 2b_5_N_max_, b_1_ = b_3_ = b_4_ = 0	
**10**	**Lower extraversion and higher neuroticism are monotonously related to higher loneliness and the effect of extraversion saturates.**	**Saturating Extraversion and Linear Neuroticism Effects Model:** **b_2_ > 0, b_3_ > 0, −b_1_ > 2b_3_E_max_,** **b_4_ = b_5_ = 0**	**2b**
11	Lower extraversion and higher neuroticism are monotonously related to higher loneliness and the effect of extraversion is exponential.	Exponential Extraversion and Linear Neuroticism Effects Model: b_2_ > 0, b_3_ < 0, −b_1_ > 2b_3_E_min_, b_4_ = b_5_ = 0	
**12**	**Lower extraversion and higher neuroticism are monotonously related to higher loneliness and the effect of neuroticism is exponential.**	**Linear Extraversion and Exponential Neuroticism Effects Model:** **b_1_ < 0, b_5_ > 0, −b_2_ < 2b_5_N_min_,** **b_3_ = b_4_ = 0**	**2c**
13	Lower extraversion and higher neuroticism are monotonously related to higher loneliness and the effect of neuroticism saturates.	Linear Extraversion and Saturating Neuroticism Effects Model: b_1_ < 0, b_5_ < 0, −b_2_ < 2b_5_N_max_, b_3_ = b_4_ = 0	
**14**	**Lower extraversion and higher neuroticism are monotonously related to higher loneliness and the effect of extraversion saturates while the effect of neuroticism is exponential.**	**Saturating Extraversion and Exponential Neuroticism Effects Model:** **b_3_ > 0, b_5_ > 0, −b_1_ > 2b_3_E_max_, −b_2_ < 2b_5_N_min_,** **b_4_ = 0**	**2d**
15	Lower extraversion and higher neuroticism are monotonously related to higher loneliness and the effect of extraversion is exponential while the effect of neuroticism saturates.	Exponential Extraversion and Saturating Neuroticism Effects Model: b_3_ < 0, b_5_ < 0, −b_1_ > 2b_3_E_min_, −b_2_ < 2b_5_N_max_, b_4_ = 0	
16	Lower extraversion and higher neuroticism are monotonously related to higher loneliness and the effects of both traits saturate.	Saturating Effects of Extraversion and Neuroticism Model: b_3_ > 0, b_5_ < 0, −b_1_ > 2b_3_E_max_, −b_2_ < 2b_5_N_max_, b_4_ = 0	
17	Lower extraversion and higher neuroticism are monotonously related to high loneliness and the effects of both traits are exponential.	Exponential Effects of Extraversion and Neuroticism Model: b_3_ < 0, b_5_ > 0, −b_1_ > 2b_3_E_min_, −b_2_ < 2b_5_N_min_, b_4_ = 0	
	*Linear interactions (mutual dependence)*		
**18**	**Lower extraversion and higher neuroticism are linearly associated with higher loneliness and both effects buffer each other.**	**Mutual Compensation Model:** **b_4_ < 0, b_1_ + b_4_N_min_ < 0, b_2_ + b_4_E_max_ > 0,** **b_3_ = b_5_ = 0**	**2e**
**19**	**Lower extraversion and higher neuroticism are linearly associated with higher loneliness and both effects reinforce each other.**	**Optimal Constellation Model:** **b_4_ > 0, b_1_ + b_4_N_max_ < 0, b_2_ + b_4_E_min_ > 0,** **b_3_ = b_5_ = 0**	**2f**

Note: In the statistical models, L_n_ denotes the outcome variable loneliness, and E_n_ and N_n_ denote the predictor variables extraversion and neuroticism, respectively. E_min_/E_max_ = minimal/maximal value of extraversion in the data, N_min_/N_max_ = minimal/maximal value of neuroticism in the data. The constraints that involve E_min_, E_max_, N_min_, and/or N_max_ ensure that the Model’s predictions are in line with the respective hypothesis for the whole range of realistic predictor values, where the empirically observed values are used as a proxy for the range that is realistic. In the longitudinal analyses, L_n_ measured at T2 served as the outcome variable and L_n_ measured at T1 was added as a control variable. Hypotheses and models in bold were included in the initial hypothesis set.
